# Native intra-articular knee microbiome is a matter of facts: a systematic review of clinical evidence

**DOI:** 10.1530/EOR-23-0191

**Published:** 2024-10-03

**Authors:** Tommaso Bonanzinga, Pietro Conte, Giuseppe Anzillotti, Vincenzo Longobardi, Elizaveta Kon, Maria Rescigno, Maurilio Marcacci

**Affiliations:** 1 IRCCS Humanitas Research Hospital, via Manzoni, Rozzano, Milan, Italy; 2 Department of Biomedical Sciences, Humanitas University, Via Rita Levi Montalcini, Pieve Emanuele, Milan, Italy; 3 Department of Traumatology, Orthopaedics and Disaster Surgery, Moscow, Russia

**Keywords:** Intraarticular, joint, knee, microbiome, microbiota, osteoarthritis

## Abstract

**Purpose:**

- Growing interest surrounds the role of human gut microbiome in the development of degenerative pathologies such as osteoarthritis (OA), but microbes have recently been detected also in other sites previously considered to be sterile. Evidence emerged suggesting that even native and osteoarthritic knee joints may host several microbial species possibly involved in the osteoarthritic degeneration. This is the first systematic review critically collecting all the available evidence on the existence and composition of knee intra-articular microbiome.

**Methods:**

- A systematic research on the PubMed, Cochrane and Google Scholar databases was performed. Human clinical studies investigating the presence of intra-articular microbiome in native osteoarthritic knee joints with next-generation sequencing techniques were collected.

**Results:**

- A total of eight studies were included reporting data on 255 knees. All the included studies reported evidence supporting the existence of an intra-articular microbiome in native knee joints, with detection rates varying from 5.8% to 100%. Bacteria from the Proteobacteria phylum were found to be among the most identified followed by the Actinobacteria, Firmicutes, Fusobacteria, and Bacteroideta phyla*.* Proteobacteria phylum were also found to be more common in osteoarthritic knees when compared to healthy joints. Furthermore, several pathways correlating those microbes to knee OA progression have been suggested and summarized in this review.

**Conclusions:**

- Evidence collected in this systematic review suggests that the native knee joint, previously presumed to be a sterile environment, hosts a peculiar intra-articular microbiome with a unique composition. Furthermore, its alteration may have a link with the progression of knee osteoarthritis.

## Introduction

Osteoarthritis (OA), most commonly developing in the knee joint, is the main cause of disability in older adults and is characterized by pain, loss of function, and decreased quality of life (QoL). Nowadays, an estimated 240 million individuals worldwide have symptomatic OA, including in 10% of men and 18% of women aged 60 and older (1). Its incidence is rapidly increasing due to an aging and obese population, which causes a huge financial burden globally, expected to be in most countries up to 2.5% of the gross domestic product (2). So far, no effective treatment has shown efficacy in reversing OA progression (3, 4, 5), with joint replacement as the only definitive choice available. This has led to an increasing number of arthroplasty procedures done worldwide despite periods of economic downturn (6) and an average age of patients undergoing joint replacement around the mid-60s, but with increasing numbers of patients younger than 60 years (7). Obviously, this exposes patients to a higher lifetime risk of revision and a not insignificant economic burden, given that in the United States alone the annual cost exceeds US$15 billion (8). Therefore, increasing interest is oriented toward a better understanding of the pathogenesis of OA, with the aim of addressing the trigger factors and implementing preventive actions to delay or slow down its progression. In consequence, as the pathogenesis and treatment of OA have not been completely elucidated, a better knowledge of the natural history of early OA will help identify new targets for intervention (9, 10).

Recently, studies have consistently revealed the fact that both local and systemic inflammations play pivotal roles in OA (11). Among others, gut microbiota is responsible for a series of metabolic, immunological, structural, and neurological functions, such as maintenance of metabolic homeostasis, development and maturation of immune system, resistance to infections, and production of neurotransmitters (12, 13). Dysbiosis, which has emerged as a hidden risk factor inducing the production of pro-inflammatory cytokines and bacterial metabolites, may boost the pathophysiological mechanisms of OA (14). Indeed, a ‘gut–joint’ axis in the development of OA has been hypothesized (15, 16) and preclinical studies on animal models support the idea that migration of pathogenic bacteria from the gut to the synovium in OA patients can contribute to the pathogenesis of this condition (17, 18). Moreover, recent evidence shows that the severity of knee joint damage and synovial inflammation in patients with OA was positively linked to high levels of lipopolysaccharide (LPS), and the production of intestinal bacteria and LPS could induce inflammation and damage in joints through macrophage activation and damage-associated molecular patterns (DAMPs) (19, 20). Thus, the products and metabolites of the gut microbiota appear to influence OA progression by activation of the immune system through different pathways (21).

A great impulse in this quest was given by the introduction of next-generation sequencing (NGS). Indeed, NGS techniques and metagenomics can provide a view of the transcriptome of the host tissue as well as capture all microbial genomes (i.e., bacteria, fungi, and viruses) present in a given sample. This has led to new opportunities to investigate the role of microbiota in sites traditionally assumed to be sterile and has allowed the finding of sequencing data from cerebrospinal fluid (22), amniotic fluid (23), and benign breast tissue (24), suggesting that a native microbial DNA signal could also be present in other sites such as native joints. In fact, there is already evidence indicating the existence of a shoulder joint microbiome (25). Furthermore, prior studies have identified bacteria in hip and knee implants requiring revision for presumed aseptic failure (26), and others, evaluating the utility of NGS for the diagnosis of periprosthetic joint infection (PJI), have identified the presence of microorganisms in 13.9%–71% of patients in the control group without PJI (27, 28, 29). To date, it is still impossible to establish if organisms found with NGS techniques in patients undergoing total knee replacement (TKR) revision for aseptic failure must be attributed to false negatives of standard diagnostic tools such as cultures, or if they are to be seen as initial evidence of the existence of a knee intra-articular microbiome. To further address this issue, NGS evaluations on native OA knee joints are needed, and several studies on this specific topic have been published in the last years. To our knowledge, to date, there is no review summarizing all this evidence; therefore, given the clear value of understanding the potential role of such a microbiome, the aim of the present review is to summarize all the current evidence on the existence of the intra-articular microbiome and its potential correlation with the development of knee OA (KOA).

## Materials and methods

The present systematic review was conducted based on the method recommended by the preferred reporting Items for systematic review and meta-analyses (PRISMA) guidelines (2020) (30) and following the PRISMA checklist indications (31). Extensive research on the PubMed, Cochrane, and Google Scholar databases was performed on July 15, 2023, using the following words: (microbiome OR microbiota OR bacteria) AND (knee osteoarthritis OR knee OA). The screening process and analysis were conducted separately by three independent observers (PC, GA, and VL). First, the articles were screened by title and abstract. The following inclusion criteria for relevant articles were used during the screening: (1) clinical trials on humans (2) written in English; and (3) dealing with the sequencing of the knee intra-articular microbiome of patients diagnosed with primary osteoarthritis. Exclusion criteria were: (1) articles written in other languages; (2) animal or *in vitro* trials; (3) reviews and meta-analyses; (4) trials on joints different from the knee; (5) trials focusing only on revision TKR not including at least an evaluation on native joints; and (6) trials dealing with articular pathologies different from primary osteoarthritis.

**Figure 1:**
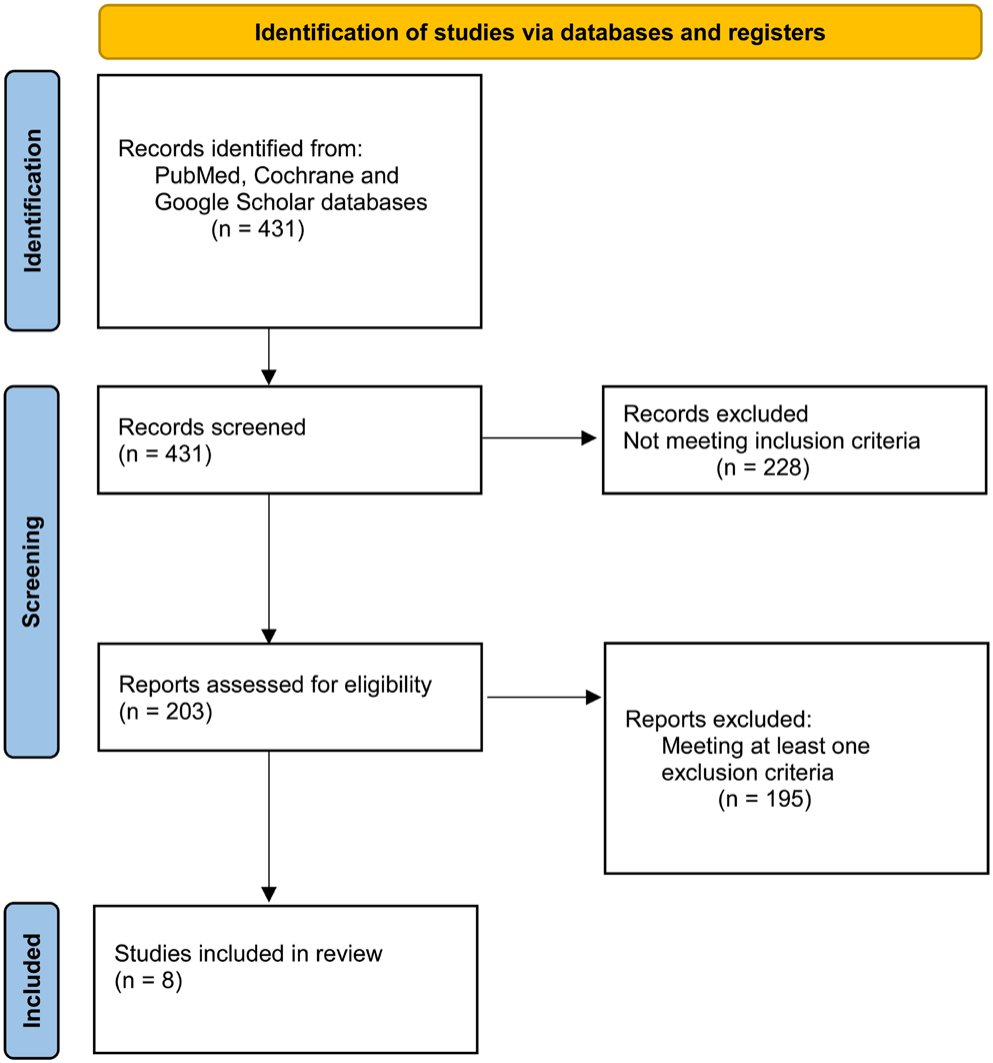
PRISMA 2020 flow diagram for the present systematic review reporting search strategy and outputs.

Secondly, the full text of the selected articles was screened according to the inclusion and exclusion criteria. Thirdly, the reference list of all the retrieved articles was further screened. A flowchart of the systematic review is provided in Fig. 1. Fourthly, discrepancies between the three reviewers were resolved by discussion and consensus, and finally, the results were approved by a senior investigator (TB and EK).

## Results

### Studies selection and characteristics

Four hundred and thirty-one studies were screened according to the inclusion and exclusion criteria. A total of eight studies were finally included in this systematic review (Table 1). All the included studies were cross-sectional evaluations, while longitudinal studies on microbiome presence and subsequent OA development are totally lacking in the present literature. Seven of the eight included studies were prospective clinical studies, whereas the study from Tsai *et al.* (32) was a retrospective case-control study in which RNA-sequencing data from synovial tissue of OA patients was aligned to a library of microbial reference genomes to identify microbial reads indicative of different microbial abundance between the two cohorts.

**Table 1:** Main features and evidence collected from the included studies on knee intra-articular microbiome.

**Authors**	**Cohort**	**Main findings**	**Additional findings**
Goswami *et al.*, 2023 (38)	113 OA patients undergoing 73 TKR and 40 THR	Positive results 96.5% of cases for a total of 578 OTUs. Five most abundant genera: *Escherichia*, *Cutibacterium*, *Staphylococcus*, *Acinetobacter*, and *Pseudomonas* that are not the ones identified in previous skin microbiome studies.	Hospital of origin explained a significant portion of the variance. Prior corticosteroid injection was associated with an elevated abundance of several lineages. Swabs yielded a slightly higher diversity compared with synovial fluid and tissue.
Torchia *et al.*, 2019 (33)	40 knee OA patients undergoing primary TKR	30% of the patients had at least one positive organism identified by NGS from their native OA knee. Average number of organisms per patient: 4.6×. *Escherichia coli* sp.,the most common in native knees.	All sterile controls were negative for any pathogen on NGS. All patients with prior knee surgeries had no organisms identified by NGS at the time of TKR. No patients sustained a PJI at the 3-month follow-up.
Zhao *et al.*, 2018 (36)	125 patients with RA and 58 with OA undergoing knee joint surgery	*Porphyromonas* and *Bacteroides* sp., in all samples (RA + OA). The most abundant phyla in all synovial samples:Proteobacteria,Bacteroidetes, and Firmicutes. The synovial tissue of patients with OA was mainly composed of* Bacteroides*,* Megacoccus*,* Haemophilus*,* Porphyromonas*,and* Streptococcus* species*,* while the synovial fluid of patients with OA was predominantly composed of* Bacteroides* sp.	An abundant diversity of bacterial nucleic acids between both RA and OA, and between synovial tissue and fluid in each pathology, was noted.
Fernández-Rodríguez *et al.*, 2023 (35)	55 patients/65 knees divided into 5 groups: 15 non-OA, 14 OA, 14 aseptic knee revision, 12 septic knee revision, 10 controlateral non-OA	77 OTUs were detected, and the highest number of species was found in native OA knees (*P* ≤ 0.035). High abundance of Proteobacteria phylum was observed in the OA joints, in the aseptic revision, and contralateral non-OA groups, but none of the OTUs observed in the study were members of the *Pseudomonas* genus. *Cutibacterium* and *Paracoccus* species were detected in all groups. *Cutibacterium*, *Staphylococcus*, and *Paracoccus* species were dominant in non-OA knees, covering nearly 75% abundance.	All the positive cultures were in the septic knee revision group, with *Staphylococcus* species being the most prevalent. Three of the patients in the PJI group had negative cultures. The NGS analysis of patients who had a PJI diagnosis, confirmed the culture results.
Tsai *et al.*, 2020 (32)	24 synovial samples: 14 osteoarthritic knees, 10 healthy controls	43 species were significantly dysregulated between OA and healthy patients. More than 50% were *Pseudomonas* genus. 9 species were found to be significantly correlated to immune signatures and OA pathways, and most of them were *Pseudomonas* genus.	299 bacterial species were identified in OA samples vs 84 in non-OA. Microbes correlated to OA are related to dysregulation of two main functional pathways: increased inflammation-induced extracellular matrix remodeling and decreased cell signaling pathways crucial for joint and immune function. The high prevalence of *Pseudomonas* genus in this cohort may be linked to existing regional differences (Australian population).
Tarabichi *et al.*, 2018 (27)	17 primary (8 TKR, 9 THR) and 65 revisions for septic and aseptic failure (39 TKR, 26 THR) arthroplasties	35.3% of primary arthroplasties negative at cultures were positive at NGS. All positive NGS samples originated from tissue, while swabs and fluid were all negative. Proteobacteria, Fusobacteria, Actinobacteria phyla were detected with high percentages in native OA joints. 25.0% of aseptic revisions with negative cultures were positive with NGS. *Propionibacterium acnes* sp. was the most frequently isolated (six out of nine) in those cases.	NGS was positive in 81.8% of culture-negative PJI. In 88.2% of culture-positive septic revisions, there was concordance with NGS. In six of these patients, NGS detected several other pathogens compared to cultures. NGS was more sensitive (89.3%) but less specific (73.0%) compared with cultures (60.7% and 97.3% respectively).
Dunn *et al.*, 2020 (34)	34 hips and 31 knees undergoing TKR/THR, cadaveric samples, mice	Non-OA cadaveric samples were more microbiologically diverse than both OA-eroded and OA-intact samples OA samples demonstrated increased gram-negative constituents: Proteobacteria phylum was particularly enriched. Knee OA samples were characterized by Firmicutes, whereas hip OA samples were enriched in Proteobacteria phylum.	Hip samples were microbiologically distinct from knee samples. Hip samples demonstrated lower alpha-diversity than knees. Increase in LPS production, phosphatidylinositol signaling, and nitrogen metabolism, and decreases in sphingolipid metabolism were associated with OA.
Témoin *et al.*, 2012 (37)	36 patients with periodontal disease and knee arthritis (OA or RA)	Bacterial DNA in 13.9% of synovial fluids: *F*.* nucleatum* sp.was detected in 80% of positive cases. The detection rate in primary KOA was 5.8% (1/17 pts).	Of the five positive cases, two were diagnosed with periodontitis and had identical bacterial clones (*F. nucleatum* and *S*.* proteamaculans* sp.) in both the synovial fluid and dental plaque samples, suggesting the possibility of infection translocating from the periodontal tissue to the synovium.

NGS; next-generation sequencing, OA; osteoarthritis, OTUs; operational taxonomic units, PJI; periprosthetic joint infection, RA; rheumatoid arthritis, THR; total hip replacement, TKR; total knee replacement

The sequencing of samples from native osteoarthritic knee joints was conducted in all the included studies: the evaluation was limited to KOA patients (33) or associated with the sequencing of healthy joints (32, 34, 35), arthritic non-OA joints (36, 37), septically and aseptically failed TKR (27, 35, 37) or hip joints (27, 34, 38).

A total of 255 native knee OA joints were sequenced in the included studies: the biggest cohorts were the ones from Goswami *et al.* (73 patients) (38), Zhao *et al.* (58 patients with OA) (36) and Torchia *et al.* (40 patients with OA) (33), while the other cohorts were particularly limited in number (8–31 patients with OA). All the included studies were published in the last 5 years, except the one from Temoin *et al.* from 2012.

Intra-articular samples generally included synovial fluid and/or synovial tissue, while tissue swabs were performed and evaluated only by Tarabichi *et al.* and Goswami *et al.* Interestingly, Temoin *et al.* considered a cohort composing only patients diagnosed with both periodontal disease and arthritis (25 OA, 11 rheumatoid arthritis) to investigate possible pathogenetic correlations and therefore performed the sequencing of both synovial fluid and periodontal plaque swabs (37). Dunn *et al.* performed a unique evaluation: eroded and intact sections of 34 hip and 24 knee cartilages were obtained from patients undergoing arthroplasty for end-stage primary OA and were compared to cadaveric OA-free cartilage controls to investigate eventual microbial signature differences in each different tissue (34).

### Evidence on OA knee intra-articular microbiome existence

Albeit limited to eight studies for a total of 255 native osteoarthritic knee joints, all the included studies reported evidence supporting the existence of an intra-articular microbiome in native osteoarthritic knee joints.

The proportion of KOA patients in which NGS detected microbes varied consistently from 5.8%, in the study by Temoin *et al.*, to 30% and 35%, in the studies by Torchia and Tarabichi *et al*, to 96.5%, in the study by Goswami *et al.* (including both knee and hip OA without a distinct evaluation), to 100%, in the study by Zhao *et al.* (27, 33, 36, 37, 38). The remaining studies did not report the proportion of samples in which NGS identified microbes (32, 34, 35). In Goswami *et al.*’s study, NGS led to positive results in 113 out of 117 joint replacements (73 TKR and 40 total hip replacement THR) for a total of 578 different operational taxonomic units (OTUs) (38). Similarly, Tsai *et al.* identified 299 different OTUs in the 14 KOA samples (versus 84 OTUs found in non-OA patients) (32), and Fernandez-Rodriguez *et al.* identified 77 OTUs in the 55 included patients (35). Furthermore, they also reported that the highest number of species was found in native OA knees when compared to non-OA knees and aseptic and septic revisions (*P* < 0.035) (35). Torchia *et al.* reported an average number of organisms identified by NGS of 4.6 per patient (33) .

As per the other evaluated cohorts, NGS identified microbes in 25% of aseptic revisions in those with negative cultures, while the detection rate in septic revisions was 81.8% in those with negative cultures and 94.1% in those with positive cultures (27). Zhao *et al.* included patients with both OA (58 patients) and rheumatoid arthritis (125 patients), and NGS identified microbes in 100% of both cohorts (36).

Sequencing of sterile control was conducted by Torchia *et al.* and no microorganism was found with NGS, thus excluding ambient contamination (33). In the other four studies, controls were collected, but results were not reported in the manuscripts (34, 35, 36, 37).

### Characterization of knee OA intra-articular microbiome

The composition and relevant abundance of each microbial species were found to be different in each included study, but some similarities still emerged from the comparison.

Bacteria from the Proteobacteria phylum were found to be among the most identified in native osteoarthritic knees. Zhao *et al.* reported that Proteobacteria was the most identified phylum in their cohort of patients with both OA (55.1% of synovial tissues and 39.1% of synovial fluids) and rheumatoid arthritis (69.0% of synovial tissues and 24.9% of synovial fluids), followed by the BacteroidetaandFirmicutes phyla (36). Dunn *et al.* identified substantial increases in the proportion of constituent microbial DNA from gram-negative organisms in OA patients compared to non-OA cadaveric controls (*P* = 0.02) and, among OA specimens, Proteobacteria phylum were particularly enriched. Generally, they reported that microbial alpha-diversity was reduced in human OA vs non-OA controls (*P* < 0.0001), and in hip samples vs knees (*P* < 0.0001), suggesting that an increase in OA progression could be linked both to a reduction in microbial diversity and to an increase in the proportion of gram-negative microbes (34).

Torchia *et al.* found *Escherichia coli* sp., again belonging to the Proteobacteria phylum, to be the most common microorganism detected in their 40 patients undergoing primary TKR (33) and the same species was among the five most abundant in the 117 joint replacements from the Goswami *et al.* study, together with *Cutibacterium*, *Staphylococcus*, *Acinetobacter*, and *Pseudomonas sp* (38). The latter emerged as relevant in Tsai *et* *al.*’s retrospective library-based study: among the 43 microbial species that were significantly dysregulated between OA and healthy patients, more than half of these were *Pseudomonas* sp.(32). On the other hand, Fernandez-Rodriguez *et al.* reported a high abundance of Proteobacteria phylum in the OA, aseptic revision, and contralateral non-OA groups but further specified that none of the OTUs observed in the study were members of the *Pseudomonas* sp.(35). This difference could be a result of the microbiome being highly dependent on geography: evidence exists that *Pseudomonas* sp. has a high prevalence in the microbiome of the Australian population as the one considered in Tsai *et al.* (39, 40) and may have a lower prevalence in other populations. Furthermore, this difference may be the result of processing different clinical samples (biopsies for Tsai *et al.* vs synovial fluid for Fernandez-Rodriguez *et al.*), which could have affected the absolute and relative abundance of different species.

The impact of geography on intra-articular microbiome was evident also in the only multicenter study included in this review, where the hospital of origin of the sample was associated with beta diversity (i.e., bacterial composition), explaining 18.5% of the observed variation among individuals (38).

The four other phyla frequently identified in native osteoarthritic knees were Actinobacteria, Firmicutes, Fusobacteria, and Bacteroideta.

Actinobacteria phylum was reported with high frequency in three studies: *Cutibacterium* sp. was in all the groups composing the cohort of Fernandez-Rodriguez *et al.*’s study (35) and the entire Actinobacteria phylum was among the dominant in native joints in the remaining studies (27, 38).

Firmicutes phylumwas generally abundant in both osteoarthritic (34, 36, 38) and non-osteoarthritic (35) knees. Indeed, *Cutibacterium, Paracoccus, and Staphylococcus* species were reported to be dominant in non-osteoarthritic knees, but the latter was also found to be the most prevalent in septic revisions with positive cultures (35) and culture-negative PJI (27). As already mentioned above, Firmicutes, along with the BacteroidetaandProteobacteria phyla*,* were the most abundant phyla in all synovial samples of the 183 patients (OA and RA) considered by Zhao *et al.* (36).

Dunn *et al.*, despite reporting an increase in gram-negative microbes in osteoarthritic samples, identified Firmicutes as a characterizing phylum for knee OA samples while hip OA samples were enriched in Proteobacteria phylum(34).

Fusobacteria phylum was among the three detected with high percentages in native joints by Tarabichi *et al.* (27) and *Fusobacterium nucleatum* sp.was found in four out of the five positive arthritic cases by Temoin *et al.* (37). Interestingly, in their study, identical bacterial clones (*F. nucleatum* and *Serratia proteamaculans* spp.) were detected in both the synovial fluid and dental plaque samples of patients diagnosed with both arthritis and periodontitis, suggesting the possibility of infection translocating from the periodontal tissue to the synovium (37).

Bacteroideta phylumwere reported as relevant only in one study: Zhao *et al*. found *Porphyromonas* and *Bacteroides* sp. in 100% (183/183) of the synovial samples they collected. Specifically, they further reported that the synovial tissue of patients with OA was mainly composed of *Bacteroides*, *Megacoccus*, *Haemophilus*, *Porphyromonas*, and *Streptococcus* spp., while the synovial fluid was predominantly composed of *Bacteroides* sp.(36).

### Microbiome presence in healthy knees

Only 35 healthy, non-arthritic knee joints were evaluated in the included works, thus limiting the generalizability of the specific findings. Nonetheless, all three studies comparing OA to non-OA patients, which aimed to find different microbial signatures between the two, found Proteobacteria phylum to be more abundant in OA, while *Cutibacterium*, *Staphylococcus*, and *Paracoccus* spp. were dominant in native non-OA knees, comprising nearly 75% of the bacterial abundance (32, 34, 35). Interestingly, Tsai *et al.* reported that not all 84 OTUs found in non-OA patients were also among the 299 OTUs found in the OA group, with only 36 overlapped (32). As already mentioned above, in their study, 43 microbial species were identified to be significantly dysregulated between OA and non-OA patients, with almost half being *Pseudomonas* sp.(32).

### Intra-articular microbiome and osteoarthritis: is there a correlation?

A possible link between extra and intra-articular microbiome and osteoarthritis was investigated by Temoin *et al.*: a cohort of 36 patients diagnosed with both periodontal disease and arthritis (25 OA and 11 rheumatoid arthritis) were evaluated by performing the sequencing of both intra-articular synovial fluid and periodontal plaques. The group reported that in five of the 36 synovial fluids (13.9%) bacterial DNA was found, and four of these five were *F*.* nucleatum* sp. Narrowing the evaluation to native knee OA patients, the detection rate was brought down to 5.8% (1/17 patients). Interestingly, *F*.* nucleatum* sp. was also found in the periodontal plaques of two of those five patients, suggesting the possibility that microbial translocation from the oral cavity to the knee joint had occurred (37). Unfortunately, the study did not include non-osteoarthritic patients; therefore, it is not possible to infer any causative connection between *F*.* nucleatum* sp.and OA development.

On the other hand, this possible causative connection was investigated in the cohort of Tsai *et al.* in which Gene Set Enrichment Analysis and the Weighted Gene Co-expression Network Analysis were conducted to correlate the expression of genes in immune signaling pathways and signatures involved in osteoarthritis development to microbes that were found to be dysregulated between OA and non-OA patients. Among those dysregulated species, nine were found to be significantly correlated to immune signatures and OA pathways, with five of those being part of *Pseudomonas* sp. The authors concluded that those microbes might play a role in increased inflammation-induced extracellular matrix remodeling and could be involved in decreased cell signaling pathways crucial for joint and immune function. More specifically, those microbes were associated with the downregulation of both neutrophil degranulation and Wnt and Rho GTPase signaling pathways, with all factors being linked to increased catabolic activity. Furthermore, those microbial species were found to have a positive correlation with activated mast cell infiltration and a negative correlation with CD8+ T cells. Although high CD8+ T cells seem to be linked to OA progression, the authors remarked on the importance of the CD4+/CD8+ ratio that has been reported to be higher in OA synovial tissue. The reported suppression of CD8+ cells by high microbial abundance could therefore lead to a skewed CD4+/CD8+ ratio, thus promoting knee OA progression (32).

Dunn *et al.* performed a functional analysis and identified several bacterial functional pathways predicted to be altered in association with OA: an increase in LPS production, phosphatidylinositol signaling, and nitrogen metabolism and decreases in sphingolipid metabolism were associated with both hip and knee OA (34).

The impact of previous procedures on intra-articular microbiome presence and diversity was investigated in two studies: Torchia *et al.* stated that all five patients who underwent prior knee surgical procedures did not have any organisms identified by NGS at the time of TKA (33) while Goswami *et al.* found prior corticosteroid injection within 6 months before arthroplasty to be associated with an elevated abundance of several lineages, with *Corynebacterium kroppenstedtii* sp. being the most common species (60% of previously injected patients) (38). Nevertheless, it is worth mentioning that those presented are exclusively cross-sectional studies, while longitudinal long-term studies on healthy patients subsequentially developing knee OA are lacking in the present literature. Studies with this specific design could help better define the real causative relationship between intra-articular microorganisms and knee OA development.

### NGS technique in knee intra-articular microbiome detection

The included studies adopted either the Illumina (4) or the IonTorrent (2) platform to perform NGS, while Goswami *et al.* (38) used both sequencing platforms because their study spanned a period during which their diagnostic laboratory switched from IonTorrent to the Illumina platform. Unfortunately, there are no studies differentiating the impact of these two specific platforms on the detection of intra-articular microbiome, thus limiting the possibility of comparing the detection rates in the included studies. Nonetheless, NGS generally appears as a precious and effective tool in detecting knee intra-articular microbiome. Tarabichi *et al.* found NGS to have a sensitivity of 89.3% and a specificity of 73% in detecting any bacteria in revision arthroplasties, which makes NGS more sensitive but less specific when compared to cultures (60.7%, 97.3% respectively) (27). In the same study, NGS and cultures had a concordance of 88.2%, while Fernandez-Rodriguez *et al.* reported that NGS confirmed the culture results in all the patients with PJI (35). Furthermore, NGS was powerful in detecting microorganisms in patients with PJI: Tarabichi *et al.* reported an 89.3% detection rate in the cases considered to be infected, 94.1% in those with positive cultures, and 81.8% in those with negative cultures diagnosed as septic using the Musculoskeletal Infection Society (MSIS) criteria (27).

A remarkable variability in the included studies was also found in the type of samples that were used to detect intra-articular microbiome, including synovial fluid, collected either percutaneously or intra-operatively, synovial tissue, and swabs. The results on their specific efficacy are contrasting; therefore, until now, it is still not possible to determine the best sample to detect intra-articular microbiome. Goswami *et al.* found swabs to outperform both synovial fluid and synovial tissue in terms of profiling capacities (38) while Tarabichi *et al.* had positive samples only from synovial tissue, whereas swabs and fluids were all negative at NGS (27).

## Discussion

The main finding of the present systematic review is the existence of a native intra-articular knee microbiome composed of different microbial phyla that can be identified with NGS procedures. Nevertheless, it is worth mentioning that, through this systematic review of the present literature, only eight studies for a total of 255 native osteoarthritic knee joints were available, thus remarking the paucity of the evidence on this specific topic and limiting the generalizability of these findings.

In recent times, interest in identifying correlations between alterations in the human microbiota and the development of osteoarthritis has grown enormously. This was recently investigated in a study systematically reviewing evidence on the gut–joint cross-talk (41): the authors, despite reporting a lack of high-quality studies on the topic, concluded that the available evidence suggests that gut microbiome dysbiosis seems to aggravate OA by enhancing the immune response, thus increasing the inflammatory state of the articular environment. Specifically, gut microbiome dysbiosis seems to induce higher levels of systemic LPS and subsequent elevation of other proinflammatory factors, such as TNF-α, IL-1b, IL-6, and c-reactive protein (CRP), thus supposedly enhancing OA progression (41). From a clinical point of view, studies have identified a clinical correlation between pain scores in OA patients, and the bacterial composition of stool samples (42, 43) and small clinical trials suggest that modification in the gut microbiome could be linked to symptomatic improvements in patients diagnosed with knee OA (44, 45).

This ‘indirect’ gut–joint cross--talk, mediated by systemic inflammatory factors, may not be the only pathway linking microbial species to the development of osteoarthritis. Indeed, with the technological advance of tools such as NGA, evidence of microbes was found in sites previously presumed to be sterile: meconium, amniotic fluid (23), cerebrospinal fluid (22), and breast tissue (24) were all found to host microbial species. Furthermore, a study from Qiu *et al.* reported the presence of a low-abundance microbiome in the rotator cuff and, potentially, in other shoulder tissues of patients undergoing primary arthroplasty surgery (25).

As for the knee joint, NGS has been proven to be a precious tool in the identification of microbes in PJIs, detecting species that traditional cultures may fail to isolate (28, 29). Furthermore, reports are emerging on NGS isolating microbes in joint replacements undergoing revisions for presumedly aseptic failure: Carr *et al.* collected 248 samples from 41 patients undergoing 21 TKR and 20 THR revisions for clinically aseptic failures (4 of 41 later found to have positive intra-operative cultures) and found a higher and differentially abundant microbial diversity in patient samples compared to that of open-air controls (46). There is still a lack of agreement on whether this must be considered an initial evidence of a true microbiome present in native knee joints (already prior to primary TKR surgery) or a clinically uninfected joint implants’ colonization. Initial evidence of a native knee intra-articular microbiome has been recently published, but a comprehensive review of these evidences is not available in the literature. For this reason, and to further clarify this topic, we performed a systematic review of the literature including all the available studies investigating the presence and composition of the native osteoarthritic knee microbiome and excluding studies that included only joint revision surgeries and articular pathologies different from primary OA.

The first evidence found on our review is the paucity of studies on the specific topic: we managed to collect only eight studies with a total of 255 native OA knee joints evaluated using sequencing techniques. This paucity raises some relevant and legitimate doubts about the generalization of the presented findings but should serve as a boost in the conduction of new, possibly longitudinal, studies. Indeed, all the included studies were cross-sectional studies that, collecting data at a single time point, can be useful in identifying initial correlations but are ineffective in ascertaining causality effects. On the other hand, longitudinal studies, which collect data at multiple time points, could better clarify the causative role of specific microbiomes found at baseline in the progressive development of knee OA. Nonetheless, all the included studies found evidence of the presence of a native intra-articular knee microbiome, with percentages of positive samples ranging from 5.8% to 100% (36, 37). When considering the largest cohorts, NGS led to positive results in 96.5% (113/117) of combined hip and knee native joints (38), 100% of the 58 osteoarthritic knee joints (36), and 30% of the 40 patients undergoing TKR for primary knee OA (33).

As for the composition of the identified microbiome, five microbial phyla were found to be dominant in native osteoarthritic knees: Proteobacteria, Actinobacteria, Firmicutes, Fusobacteria, and Bacteroideta. The Proteobacteria phylumwas the most identified in the majority of the studies (33, 34, 35, 36) and an increase in gram-negative constituents in the microbial DNA of OA joints was generally reported. Among Proteobacteria, *E. coli* sp. (33) and *Pseudomonas* sp. (32) were frequently identified. Three studies included both osteoarthritic and non-osteoarthritic knee joints and tried to compare the identified microbiomes to evaluate if specific microbes could be present exclusively in OA patients, thus suggesting a possible pathogenetic link (32, 34, 35). It is worth mentioning that the total amount of native, healthy, non-OA joints in the included studies was limited to 25 joints in addition to 10 cadaveric samples. Nonetheless, all three studies found Proteobacteria phylumto be more abundant in OA, while *Cutibacterium*, *Staphylococcus*, and *Paracoccus* spp.were dominant in native non-OA knees, comprising nearly 75% of the bacterial abundance in the study by Fernandez-Rodriguez *et al.* (32, 34, 35). An interesting study from Hammad *et al.*, which was not included in the present review since it focused on patients diagnosed with rheumatoid arthritis, similarly found that the microbiome of nine healthy controls was predominated by Proteobacteria (83.5%) and Firmicutes (16.1%) and, to a much lesser extent, the Actinobacteria (0.2%) and Bacteroideta (0.1%) phyla (47). Those evidences agree with the present literature reporting Proteobacteria phylum as a major constituent of the gut microbiome (48, 49) and its expansion as a possible actor in organ dysfunction (50).

On the other hand, Firmicutes phylum, also known to be constituents of the normal gut microbiome (51), was generally abundant in both osteoarthritic (34, 36, 38) and non-osteoarthritic (35) knees. Dunn *et al.* further found the Firmicutes phylumto bea characterizing phylum for knee OA samples while hip OA samples were enriched in Proteobacteria phylumand reported that microbial alpha-diversity was generally reduced in human OA vs non-OA controls (*P* < 0.0001) (34) and this reflects the reduction in microbiome diversity seen in other rheumatic diseases (52, 53, 54).

Three of the eight included studies tried to further evaluate how the intra-articular microbiome could impact OA induction and progression. Temoin *et al.* found identical bacterial clones (*F*.* nucleatum and S*.* proteamaculans* sp.) in both the synovial fluid and dental plaque of patients diagnosed with both periodontitis and knee osteoarthritis, suggesting a translocation of the bacteria from the oral cavity to the knee joint (37). Tsai and colleagues found that 43 microbial species were significantly dysregulated between OA and healthy patients and further determined specific functional patterns related to those dysregulated species (32): the two main pathways that were found to be targeted were decrease in cell signaling pathways crucial for joint and immune function and an increase in inflammation-induced extracellular matrix remodeling and catabolism, supporting the preclinical literature that reports that microbes seem to enhance the joint catabolism in OA pathogenesis (55). Lastly, Dunn *et al.* performed a functional analysis in both non-OA and OA samples. In the latter, found several bacterial functional pathways previously reported to be altered in similar conditions, such as the increase in LPS production, phosphatidylinositol signaling and nitrogen metabolism, and decreases in sphingolipid metabolism (34).

It is worth mentioning that this research has some intrinsic limitations. First, we chose to include only studies evaluating patients with native knee osteoarthritis in which intra-articular samples had been sequenced to detect microbes. This led to the exclusion of several studies including exclusively TKR revision or other rheumatic diseases that have nonetheless been mentioned above in the text. This further led to a restricted number of included studies (eight) and knee joints (255), thus limiting the generalization of the presented results. The generalizability of these results is additionally limited by the relevant methodological variation found in the included studies in terms of designs, cohorts, sample collection, and sequencing techniques. Secondly, it is worth mentioning that NGS detection does not prove the presence of live bacteria or causation of disease, and therefore clinical studies with this specific aim are required. Furthermore, no longitudinal, long-term studies on healthy patients were found in the present literature. This prevents us from clearly identifying the real cause–effect relationship between the intra-articular microbiome and subsequent knee OA development. Lastly, contamination cannot be definitively ruled out in the majority of the included studies, given that only one reported the NGS evaluation of sterile controls (33).

Future research will have to deal with these limitations. Specifically, further evidence is needed from bigger cohorts of arthritic and non-arthritic joints and mainly from longitudinal studies on healthy patients at risk of knee OA in order to better define the impact of different microbial species in the development of knee osteoarthritis. Subsequently, future research should then focus on interventional strategies targeted at microbiome modifications with the aim of possibly altering the osteoarthritic degeneration.

## Conclusion

Although limited to eight studies, initial evidence of intraarticular microbiome was found in both healthy and OA native knees. Further studies will be needed to further support this evidence and to assess the possible correlation between such presence and the onset and evolution of OA disease. We believe that those presented could be precious references inspiring researchers to perform new studies on this relevant topic.

## ICMJE Conflict of Interest Statement

All the authors declare that there is no conflict of interest that could be perceived as prejudicing the impartiality of the research reported.

## Funding statement

This work was partially supported by ''Ricerca Corrente'' funding from Italian Ministry of Health to IRCCS Humanitas Research Hospital as part of the Keystone project, grant number GR-2019-12371158.

## Author contribution statement

TB conceived the study, developed the research protocol, and coordinated the study. PC, GA, and VL performed the literature search, screened the manuscripts, and performed data abstraction. TB, PC, GA, and VL prepared the first manuscript draft. EK, MR, and MM finally contributed to final edits and critical revisions prior to submission. All the authors gave their approval to the final version of the present manuscript.
